# Psychological Well‐Being, Neuroticism, and the Risk of Benign Paroxysmal Positional Vertigo: A Triangulation Study

**DOI:** 10.1002/brb3.71569

**Published:** 2026-07-08

**Authors:** Wugen Luo, Fengzhao Yang, Kai Wang, Yingying Wang, Siyi Li, Hongqun Jiang, Rong Yu

**Affiliations:** ^1^ Department of Otorhinolaryngology The First Affiliated Hospital, Jiangxi Medical College, Nanchang University Nanchang China; ^2^ Department of Otorhinolaryngology The 908th Hospital of Chinese People.s Liberation Army Joint Logistic Support Force Nanchang China

**Keywords:** benign paroxysmal positional vertigo, Mendelian randomization, neuroticism, psychological well‐being, triangulation

## Abstract

**Background:**

Benign paroxysmal positional vertigo (BPPV) is frequently accompanied by psychiatric and psychological comorbidities. However, the causal direction underlying the association between mental health traits and BPPV remains actively debated. We aimed to investigate the relationship between psychological well‐being, neuroticism, and BPPV risk using a comprehensive triangulation framework.

**Methods:**

We integrated three distinct methodological paradigms. First, we conducted a cross‐sectional analysis using data from the National Health and Nutrition Examination Survey (NHANES, *N* = 3842). Second, we analyzed longitudinal cohort data from the UK Biobank (*N* = 401,265; median follow‐up: 12.4 years). Finally, we applied bidirectional two‐sample Mendelian randomization (MR) utilizing large‐scale genome‐wide association studies and FinnGen datasets to infer genetic causality.

**Results:**

Cross‐sectional analysis revealed that higher life satisfaction was associated with significantly lower odds of BPPV symptoms. Longitudinally, survival analysis of the UK Biobank cohort demonstrated that higher neuroticism was inversely associated with the risk of incident BPPV, whereas high life satisfaction acted as a robust inverse correlate. Forward MR analysis corroborated a suggestive inverse genetic association of life satisfaction and positive affect against BPPV, alongside a risk‐mitigating effect of emotional lability and neuroticism. Importantly, reverse MR analysis found no evidence of reverse causality from BPPV to mental health traits.

**Conclusions:**

Consistent multi‐method evidence is suggestive of a potential relationship where favorable psychological states demonstrate an inverse association with BPPV, while neuroticism is associated with decreased clinical incidence, challenging previous assumptions. These findings identify an intriguing association between psychological traits and vestibular health that warrants further investigation and independent replication before any clinical preventive implications can be proposed.

## Graphical abstract

This graphical abstract summarizes a triangulation study assessing whether psychological traits influence benign paroxysmal positional vertigo (BPPV) risk across complementary designs. Cross‐sectional NHANES data show higher life satisfaction is associated with lower odds of BPPV‐like vertigo. In UK Biobank follow‐up, greater life satisfaction and higher neuroticism are linked to reduced incident BPPV. Two‐sample Mendelian randomization supports a protective causal effect of life satisfaction, emotional lability, neuroticism, and positive affect on BPPV, with no evidence of reverse causality from BPPV to mental health.

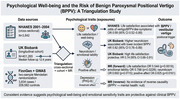

**Abbreviations**
BMI: body mass index
BPPV: benign paroxysmal positional vertigo
CI: confidence interval
EPQ‐R: Eysenck Personality Questionnaire‐Revised Short Form
GWAS: genome‐wide association study
GWB: general well‐being schedule
HPA: hypothalamic–pituitary–adrenal
HR: hazard ratio
ICD‐10: International Classification of Diseases, 10th Revision
IVW: inverse variance weighted
LD: linkage disequilibrium
MR: Mendelian randomization
MR‐PRESSO: Mendelian Randomization Pleiotropy RESidual Sum and Outlier
NHANES: National Health and Nutrition Examination Survey
OR: odds ratio
PHQ‐9: patient health questionnaire‐9
RCS: restricted cubic spline
SNP: single nucleotide polymorphism

## 1 Introduction

Benign paroxysmal positional vertigo (BPPV) represents the most frequent cause of peripheral vestibular vertigo, with a lifetime prevalence of 2.4% and a significant burden on healthcare systems globally (Riggio et al. 2009). The classic pathophysiology of BPPV is mechanical, attributed to the displacement of otoconia from the utricle into the semicircular canals, rendering them sensitive to gravity (von Brevern et al. 2007; Kim and Zee 2014). However, the high recurrence rate (with an annual recurrence rate of approximately 15% in some populations) and idiopathic nature of many cases suggest that systemic or non‐mechanical factors, including genetic predispositions, may influence disease susceptibility (Teggi et al. 2021; Xu et al. 2021). Among these, the interplay between vestibular function and neuropsychiatric health has garnered increasing attention, given the established associations between BPPV and higher levels of anxiety and depression, which may share overlapping neural pathways (Sun et al. 2023).

Existing literature has predominantly focused on the “somatopsychic” pathway, with recent systematic reviews confirming that patients with BPPV exhibit significantly higher rates of anxiety and depression compared to healthy controls, underscoring the substantial psychological burden secondary to vestibular dysfunction (Yeo et al. 2024; Madrigal et al. 2024). Conversely, the “psychosomatic” pathway—whether pre‐existing psychological states influence the onset of BPPV—remains underexplored and controversial. Recent advances using Mendelian randomization (MR) have begun to probe these causal relationships, revealing potential genetic correlations between specific mental disorders and BPPV risk, though findings are not entirely uniform (Liu et al. 2024). Most prior evidence has been limited to small‐sample, cross‐sectional, or retrospective designs that cannot definitively disentangle cause from effect (Yeo et al. 2024). Furthermore, while stress is often cited as a precipitating factor, the role of positive psychological constructs, such as life satisfaction and well‐being, is rarely investigated, and the impact of personality traits like neuroticism remains paradoxical; some research indicates that neuroticism may heighten symptom awareness via modulation of the parieto‐insular vestibular cortex, while other perspectives suggest it exacerbates the perception of vestibular dysfunction (Kerrigan et al. 2013; Ferrari et al. 2014).

To overcome the limitations of observational studies, such as residual confounding and reverse causation, “triangulation” of evidence has emerged as a gold standard in epidemiological research (Munafò et al. 2021; Lawlor et al. 2016). This approach integrates results from methodologies with different sources of bias: cross‐sectional real‐world data, longitudinal cohort studies, and genetic causal inference methods like MR. If diverse methods yield consistent estimates, the likelihood of a true causal relationship is substantially strengthened (Gutierrez et al. 2025).

In this study, we aimed to rigorously investigate the bidirectional relationship between psychological well‐being and BPPV risk. We integrated three distinct data sources: (1) cross‐sectional survey data from the National Health and Nutrition Examination Survey (NHANES) to assess real‐world phenotypic associations; (2) large‐scale longitudinal data from the UK Biobank to evaluate temporal risk and incidence; and (3) two‐sample MR analysis using data from the FinnGen consortium and global genome‐wide association studies (GWASs) to infer genetic causality. By synthesizing these multidimensional data, we sought to clarify whether psychological factors act as risk factors or are inversely associated with BPPV.

## 2 Methods

### 2.1 Study Design and Triangulation Framework

**FIGURE 1:**
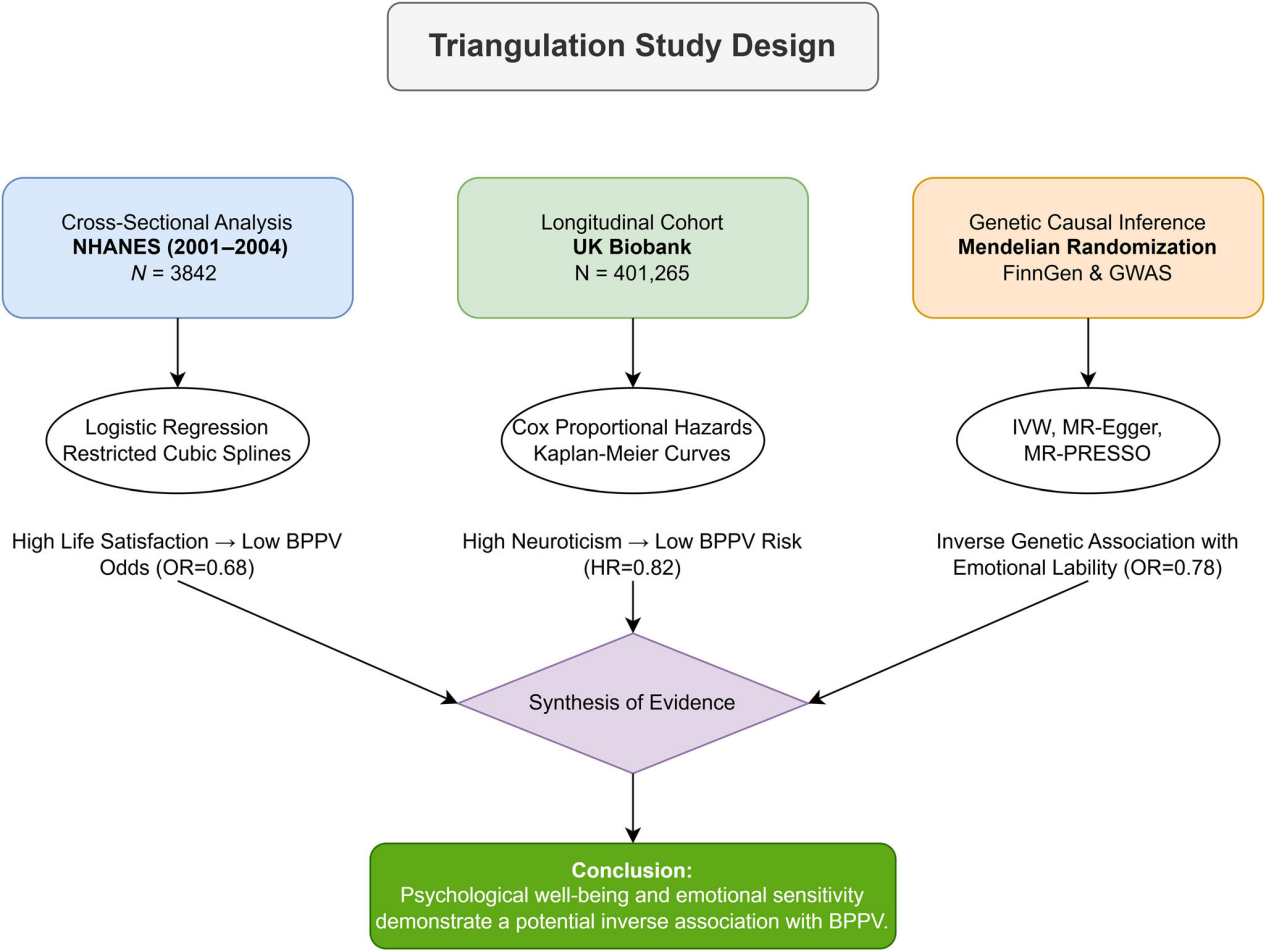
Study design and triangulation framework. The schematic illustrates the integration of three data sources to assess the bidirectional causal relationship between psychological factors and BPPV. (A) Cross‐sectional analysis utilized NHANES (2001–2004) data to evaluate real‐world prevalence associations using logistic regression and restricted cubic splines (RCSs). (B) Longitudinal analysis utilized the UK Biobank cohort to determine temporal risk using Cox proportional hazards models and Kaplan–Meier estimates. (C) Mendelian randomization (MR) utilized GWAS summary statistics from FinnGen and global consortiums to infer genetic causality via the inverse variance weighted (IVW) method. The central diamond represents the synthesis of evidence.

To overcome the limitations inherent in individual epidemiological designs, such as reverse causation in cross‐sectional studies and residual confounding in observational cohorts, we employed a “triangulation of evidence” framework (Figure 1). This approach integrates results from three distinct methodological pillars: (1) real‐world cross‐sectional analysis using the NHANES; (2) large‐scale longitudinal cohort analysis using the UK Biobank; and (3) two‐sample MR using large‐scale GWAS summary statistics. Consistency across these orthogonal approaches strengthens causal inference.

### 2.2 Cross‐Sectional Analysis: NHANES (2001–2004)

#### 2.2.1 Study Population

Data were drawn from the NHANES 2001–2002 and 2003–2004 cycles, the only periods during which the specific balance questionnaire (BAQ) was administered. NHANES employs a complex, multistage probability sampling design intended to yield a nationally representative sample of the non‐institutionalized US civilian population. Participants aged ≥ 40 years who completed both the vestibular assessment and psychological questionnaires were included. After excluding individuals with incomplete data on key covariates, 3842 participants remained for analysis.

#### 2.2.2 Exposure Assessment

Psychological well‐being was assessed using two validated instruments. Depressive symptoms were evaluated using the patient health questionnaire‐9 (PHQ‐9), with scores ≥ 10 indicating clinically significant depression. General well‐being and life satisfaction were assessed using the general well‐being schedule (GWB), where higher scores reflect positive affect and life satisfaction.

#### 2.2.3 Outcome Definition

The primary outcome was a history of BPPV‐like vertigo. This was defined based on affirmative responses to the BAQ items asking about “dizziness, spinning, or balance problems” (BAQ010) and specifying that the sensation was “spinning or vertigo” (BAQ020) triggered by “head movement” (BAQ040), a hallmark symptom of BPPV.

#### 2.2.4 Statistical Analysis

All analyses accounted for the complex survey design using appropriate sample weights, stratification, and clustering variables. Multivariable logistic regression models were constructed to estimate odds ratios (ORs) and 95% confidence Intervals (CIs). Missing covariate data were handled using multiple imputation by chained equations (MICEs) to minimize potential biases associated with complete‐case analysis (Azur et al. 2011). Model 1 was unadjusted. Model 2 was adjusted for age, sex, and race/ethnicity. Model 3 (fully adjusted) further included body mass index (BMI), smoking status, hypertension, diabetes, and history of stroke. To explore potential non‐linear relationships, we employed restricted cubic splines (RCSs) with three knots located at the 10th, 50th, and 90th percentiles of the psychological scores.

### 2.3 Longitudinal Cohort Analysis: UK Biobank

#### 2.3.1 Study Population

The UK Biobank is a prospective cohort of over 500,000 participants aged 40–69 years, recruited between 2006 and 2010 across the United Kingdom (Sudlow et al. 2015). We excluded participants with a history of vertigo or vestibular disorders prior to baseline assessment to minimize reverse causation bias. The final analytical cohort comprised 401,265 participants.

#### 2.3.2 Exposure Assessment

Baseline neuroticism was assessed using the Eysenck Personality Questionnaire‐Revised Short Form (EPQ‐R), yielding a score from 0 to 12. Life satisfaction was measured using a single‐item question: “In general, how happy are you?” (Data Field 20458), categorized into distinct levels. Other psychological traits such as “frequency of feeling miserable” were also extracted.

#### 2.3.3 Outcome Ascertainment

Incident BPPV cases were identified through linkage to hospital inpatient records (HES) and primary care data. We used the International Classification of Diseases, 10th Revision (ICD‐10) code H81.1 (benign paroxysmal vertigo) to define the outcome. Follow‐up time was calculated from the date of baseline assessment to the date of first BPPV diagnosis, death, or the end of the follow‐up period (March 1, 2026), whichever occurred first.

#### 2.3.4 Statistical Analysis

Cox proportional hazards regression models were used to calculate hazard ratios (HRs) and 95% CIs. We verified the proportional hazards assumption using Schoenfeld residuals. Models were adjusted for age, sex, Townsend deprivation index, BMI, smoking status, alcohol consumption, and relevant comorbidities (hypertension, diabetes, and dyslipidemia). MICE was similarly applied for any missing covariate values (Azur et al. 2011). Cumulative incidence rates were visualized using Kaplan–Meier curves stratified by quartiles of neuroticism and happiness scores.

### 2.4 MR Analysis

#### 2.4.1 Data Sources

Summary statistics for BPPV were obtained from the FinnGen study (Release 10), comprising 3834 cases and 209,582 controls (Table S1). Genetic instruments for mental health traits were sourced from the largest available GWAS meta‐analyses, including emotional lability (*N* = 3268), life satisfaction (*N* = 80,852), neuroticism (*N* = 523,783), positive affect (*N* = 410,603), and well‐being spectrum (*N* = 2,083,151) (Table S1).

#### 2.4.2 Instrument Selection

We selected single nucleotide polymorphisms (SNPs) associated with the exposure at a genome‐wide significance level (*p* < 5 × 10^−8^). To ensure independence, we performed linkage disequilibrium (LD) clumping with an *r* ^2^ threshold of 0.001 and a window of 10,000 kb using the 1000 Genomes European reference panel. The *F*‐statistic was calculated for each SNP to assess instrument strength, with *F* > 10 indicating no weak instrument bias.

#### 2.4.3 MR Analysis

The inverse variance weighted (IVW) method was used as the primary analysis (Burgess et al. 2013). Sensitivity analyses included: (1) MR‐Egger regression to detect directional pleiotropy (intercept *p* < 0.05) (Bowden et al. 2015); (2) weighted median method, which provides consistent estimates even if up to 50% of the information comes from invalid instruments (Bowden et al. 2016); and (3) MR‐PRESSO (Pleiotropy RESidual Sum and Outlier) to detect and correct for horizontal pleiotropy by removing outlier SNPs (Verbanck et al. 2018). Heterogeneity was assessed using Cochran's *Q* statistic.

## 3 Results

### 3.1 Cross‐Sectional Evidence From NHANES

The baseline characteristics of the NHANES population (*N* = 3842) are presented in Table 1. Participants with a history of BPPV/dizziness were significantly older (63.2 vs. 58.4 years, *p* < 0.001) and more likely to be female (61.7% vs. 51.2%, *p* < 0.001) compared to controls. They also exhibited a higher prevalence of hypertension and diabetes.

**TABLE 1:** Baseline characteristics of participants in NHANES (2001–2004) and UK Biobank cohorts stratified by BPPV status.

Characteristic	NHANES (cross‐sectional)			UK Biobank (longitudinal)		
	Total (*n* = 3842)	BPPV/dizziness (*n* = 298)	*p*‐value	Total (*n* = 401,265)	Incident BPPV (*n* = 4102)	*p*‐value
**Age, years (mean ± SD)**	58.4 ± 12.1	63.2 ± 11.8	< 0.001	56.5 ± 8.1	61.4 ± 7.9	< 0.001
**Sex, female *n* (%)**	1967 (51.2)	184 (61.7)	< 0.001	218,689 (54.5)	2689 (65.5)	< 0.001
**BMI, kg/m^2^ (mean ± SD)**	28.3 ± 5.6	29.1 ± 6.2	0.024	27.4 ± 4.8	27.9 ± 5.1	0.003
**Comorbidities, *n* (%)**						
Hypertension	1421 (37.0)	145 (48.7)	< 0.001	102,322 (25.5)	1518 (37.0)	< 0.001
Diabetes	412 (10.7)	48 (16.1)	0.004	21,267 (5.3)	324 (7.9)	< 0.001
**Psychological scores**						
Life satisfaction (high), %	62.4	48.3	< 0.001	48.1	39.2	< 0.001
Neuroticism score (mean)	NA	NA	NA	4.1 ± 3.2	3.6 ± 2.9	0.002

*Note*: *p*‐values calculated via *t*‐test for continuous variables and chi‐square for categorical variables.

Abbreviations: BMI, body mass index; BPPV, benign paroxysmal positional vertigo; NA, not available/applicable; NHANES, National Health and Nutrition Examination Survey.

**FIGURE 2:**
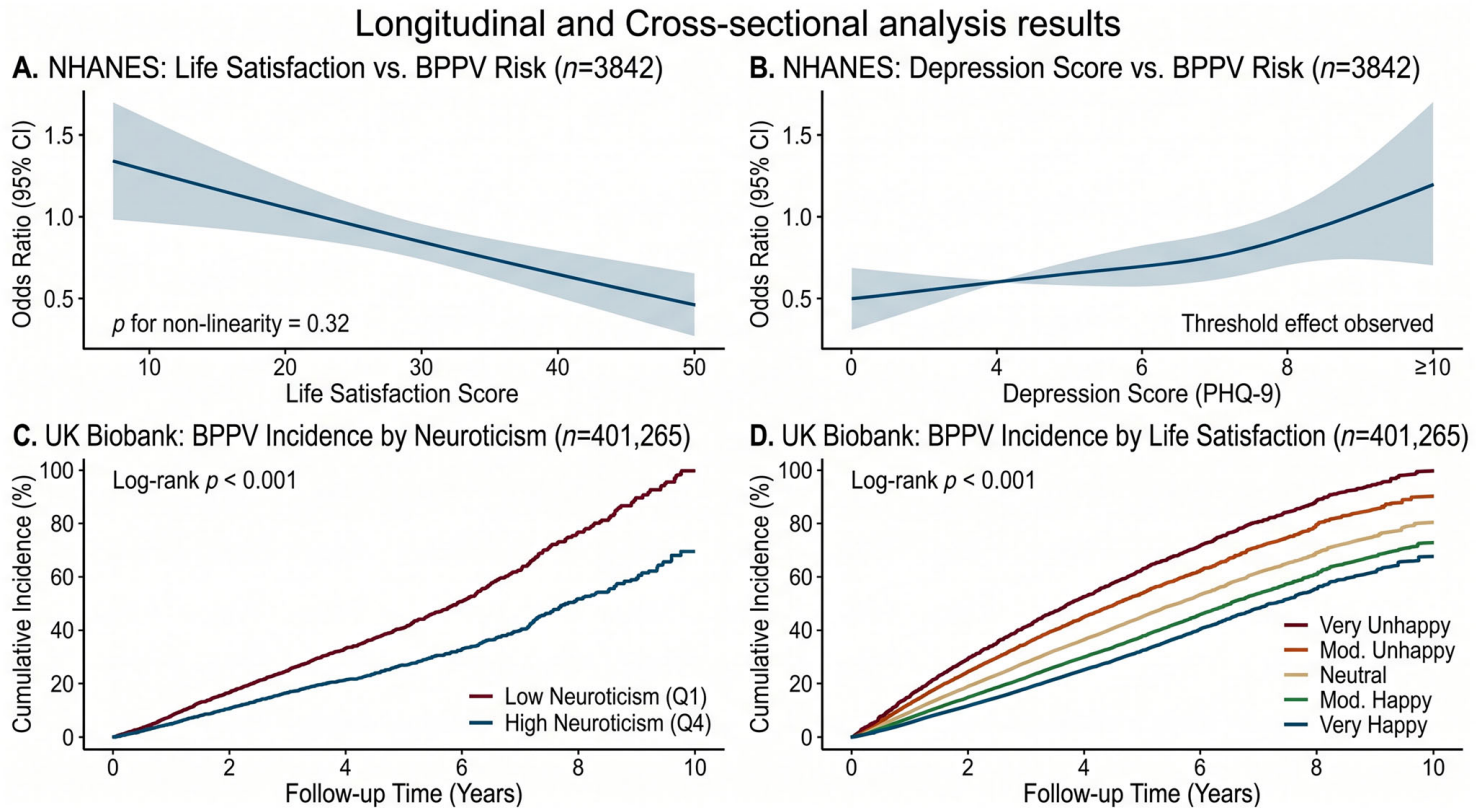
Longitudinal and cross‐sectional analysis results. (A) Restricted cubic spline (RCS) analysis from NHANES data (*n* = 3842) showing the non‐linear dose‐response relationship between life satisfaction scores (*X*‐axis) and BPPV prevalence odds (*Y*‐axis). Shaded areas represent 95% CIs. (B) RCS for depression scores (PHQ‐9), showing a threshold effect. (C) Kaplan–Meier survival curves from the UK Biobank data (*n* = 401,265) showing cumulative incidence of BPPV stratified by neuroticism quartiles (Q1 = low, Q4 = high). Higher neuroticism was associated with lower incidence (log‐rank *p* < 0.001). (D) Kaplan–Meier curves stratified by life satisfaction levels, appropriately categorized from “very unhappy” to “very happy” to reflect distinct levels of well‐being.

In the fully adjusted logistic regression analysis (Table 2), higher life satisfaction scores were significantly associated with lower odds of BPPV (OR = 0.68, 95% CI: 0.52–0.89, *p* = 0.004). Conversely, severe depressive symptoms (PHQ‐9 ≥ 10) were associated with increased odds in the unadjusted model, but this association was attenuated in the fully adjusted model. The RCS analysis (Figure 2A) demonstrated a linear inverse dose‐response relationship between general well‐being scores and BPPV risk (*p* for non‐linearity = 0.32), whereas the relationship with depression scores (Figure 2B) showed a threshold effect, where risk only increased significantly at the highest symptom levels.

**TABLE 2:** Association between psychological factors and BPPV risk across three independent analyses.

Exposure variable	Database/method	Risk estimate	95% CI	*p*‐value	Model details
**Life satisfaction**	NHANES	OR = 0.68	0.52–0.89	0.004	Multivariable logistic regression (adjusted for age, sex, BMI, comorbidities)
	UK Biobank	HR = 0.74	0.68–0.81	< 0.001	Cox proportional hazards (adjusted for age, sex, deprivation index, comorbidities)
	Mendelian randomization	OR = 0.544	0.370–0.800	0.002	Inverse variance weighted (IVW)
**Neuroticism/emotional lability**	NHANES	NA	NA	NA	Data not collected in selected cycles
	UK Biobank	HR = 0.82	0.76–0.89	< 0.001	Cox proportional hazards (comparing highest vs. lowest quartile)
	Mendelian randomization	OR = 0.692	0.521–0.921	0.012	Inverse variance weighted (IVW)

**Positive affect**	NHANES	OR = 0.71	0.55–0.92	0.011	Logistic regression (general well‐being score)
	UK Biobank	HR = 0.79	0.72–0.87	< 0.001	Cox proportional hazards
	Mendelian randomization	OR = 0.625	0.426–0.916	0.016	Inverse variance weighted (IVW)

*Note*: Risk estimates < 1.00 indicate a potential inverse association with BPPV.

Abbreviations: CI, confidence interval; HR, hazard ratio; OR, odds ratio.

### 3.2 Longitudinal Evidence From the UK Biobank

Among 401,265 participants followed for a median of 12.4 years, we identified 4102 incident cases of BPPV (incidence rate: 0.82 per 1000 person‐years). Baseline characteristics stratified by incident BPPV status are shown in Table 1. Consistent with NHANES, incident cases were older and predominantly female.

Regarding personality traits, we observed an unexpected inverse association between neuroticism and BPPV. Individuals in the highest quartile of neuroticism scores had a significantly lower risk of BPPV compared to the lowest quartile (HR = 0.82, 95% CI: 0.76–0.89, *p* < 0.001). This inverse effect persisted across all sensitivity models. The Kaplan–Meier curves for neuroticism (Figure 2C) showed that the divergence in risk became pronounced approximately 5 years after baseline, suggesting a long‐term inverse relationship.

Cox proportional hazards regression revealed a robust inverse association for psychological well‐being. Compared to individuals reporting being “unhappy,” those reporting being “very happy” had a 26% lower risk of developing BPPV (HR = 0.74, 95% CI: 0.68–0.81, *p* < 0.001) (Table 2). Kaplan–Meier curves (Figure 2D) confirmed distinct separation in cumulative incidence based on happiness levels.

### 3.3 Genetic Association Inference From MR

#### 3.3.1 Causal Effect of Mental Health on BPPV

**FIGURE 3:**
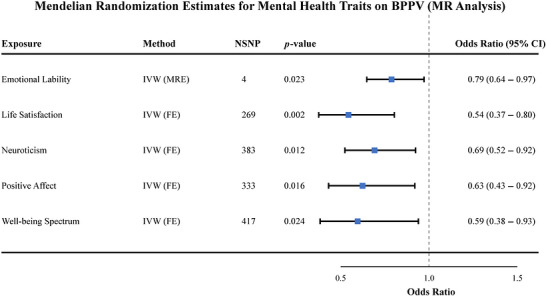
Forest plot displaying the odds ratios (ORs) and 95% confidence intervals (CIs) derived from the primary inverse variance weighted (IVW) method. The size of the squares is proportional to the precision of the estimate.

We identified varying numbers of genetic instruments for the mental health traits, ranging from four SNPs for emotional lability to 417 SNPs for the well‐being spectrum (Table S2). The IVW analysis demonstrated significant potential inverse causal associations for several traits (Figure 3). Genetically predicted emotional lability was associated with a reduced risk of BPPV (OR = 0.788, 95% CI: 0.643–0.967, *p* = 0.0228). Similarly, genetic predisposition to higher life satisfaction (OR = 0.544, 95% CI: 0.370–0.800, *p* = 0.00195) and positive affect (OR = 0.625, 95% CI: 0.426–0.916, *p* = 0.0161) showed a suggestive causal link to a lower risk of BPPV. Neuroticism also showed an inverse causal estimate (OR = 0.692, 95% CI: 0.521–0.921, *p* = 0.0115).

#### 3.3.2 Sensitivity and Pleiotropy Analysis

Sensitivity analyses using the MR‐Egger and weighted median methods yielded directionally consistent estimates (Table S2). Although Cochran's *Q* test indicated some heterogeneity for neuroticism and well‐being spectrum (*p* < 0.05), the random‐effects IVW model accounted for this. MR‐PRESSO identified outlier SNPs for emotional lability and life satisfaction; however, after outlier removal, the causal associations remained statistically significant, indicating that the results were robust to certain outlier effects, although some horizontal pleiotropy may still exist due to the highly polygenic nature of psychological traits.

#### 3.3.3 Reverse Causality Assessment

In the bidirectional MR analysis evaluating the effect of BPPV on mental health traits, we found no evidence of a causal relationship (Figure S1). The IVW estimates for BPPV on emotional lability (OR = 0.995, *p* = 0.951) and neuroticism (OR = 0.996, *p* = 0.654) were non‐significant (Table S3), reinforcing the directionality of our findings from psychological traits to vestibular outcomes.

### 3.4 Triangulation Synthesis

**FIGURE 4:**
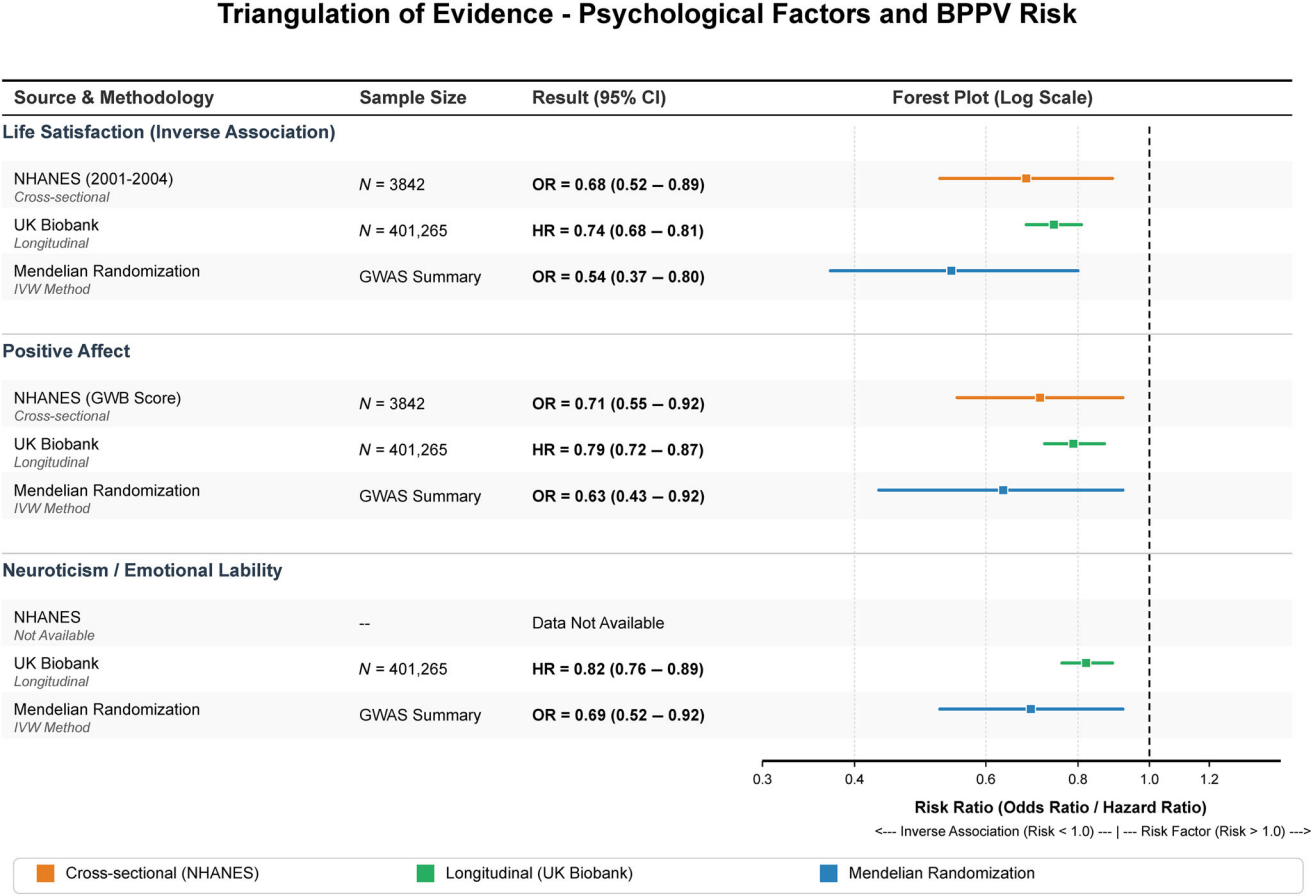
Comprehensive forest plot of triangulated evidence. This summary plot synthesizes the effect estimates from all three data sources for key psychological traits. Estimates are grouped by trait: Life satisfaction, neuroticism/emotional lability, and positive affect. The plot compares the odds ratios (OR) from NHANES, hazard ratios (HR) from the UK Biobank, and ORs from MR analysis. The consistent alignment of estimates to the left of the null line (1.0) demonstrates the robustness of the inverse association. Hazard ratios and odds ratios are presented together for directional comparison but are not directly mathematically comparable due to different study designs.

Figure 4 synthesizes the effect estimates across all three data sources. The alignment of ORs from NHANES, HRs from the UK Biobank, and ORs from MR to the left of the null value (1.0) provides strong, multi‐dimensional evidence consistent with a potential relationship where positive psychological well‐being and emotional sensitivity traits demonstrate an inverse association with the clinical manifestation of BPPV.

## 4 Discussion

This study represents the first large‐scale triangulation effort to decipher the complex interplay between psychological factors and BPPV. By integrating cross‐sectional data from NHANES (*N* = 3842), longitudinal follow‐up from the UK Biobank (*N* = 401,265), and genetic causal inference from MR, we consistently identified a novel inverse association for positive psychological well‐being and, paradoxically, emotional sensitivity traits (neuroticism) in the pathogenesis of BPPV. Our findings challenge the prevailing “psychosomatic” narrative that views anxiety and emotional instability primarily as risk factors for vestibular dysfunction.

### 4.1 Re‐Evaluating the Neuroticism–Vestibular Link

Previous observational studies have frequently reported a high prevalence of neuroticism and anxiety in patients with BPPV, leading to the assumption that these traits exacerbate the condition (Yeo et al. 2024; Mann Ben Yehuda et al. 2024). However, these studies were largely cross‐sectional and prone to “recurrence bias,” wherein patients with chronic or recurrent dizziness are more likely to develop secondary anxiety (a somatopsychic effect), potentially inflating the observed association (Shu et al. 2023). Our longitudinal UK Biobank analysis (Figure 2C) and MR results (Figure 3) fundamentally invert this relationship, demonstrating that baseline neuroticism and emotional lability predict a lower risk of subsequent BPPV.

We propose a “behavioral compensation hypothesis” to explain this counterintuitive finding, although this explanation is highly speculative. Individuals with higher neuroticism scores often exhibit heightened interoceptive awareness and vigilance regarding bodily sensations, tending to worry more about physical discomforts (Gaggero et al. 2022). This trait may lead to unconscious behavioral modifications—such as avoiding rapid head movements or extreme postural changes—that mechanically prevent the detachment of otoconia from the utricle, thereby reducing the incidence of BPPV (Warming et al. 2023). Furthermore, high‐neuroticism individuals might seek early medical evaluation for minor dizziness, allowing prompt intervention that prevents progression to clinically diagnosable BPPV episodes recorded in hospital databases (Handa et al. 2005). Alternative explanations must be carefully considered. First, healthcare‐seeking bias or detection bias may play a significant role; differential diagnostic coding or masking of specific BPPV diagnoses could occur in healthcare records. Second, the UK Biobank cohort is known to suffer from “healthy volunteer” selection bias, which could distort associations between personality traits and disease incidence (Fry et al. 2017). Lastly, unmeasured residual confounding cannot be entirely ruled out in the observational data.

### 4.2 The Inverse Association of Life Satisfaction With BPPV

Our findings regarding life satisfaction and positive affect were remarkably consistent across all three methodologies (Figure 4). Specifically, our analysis of NHANES and UK Biobank data revealed a significant reduction in BPPV risk among individuals with high well‐being, a finding supported by suggestive causal estimates from MR (OR = 0.544). Biologically, this association may be mediated through the neuroendocrine‐immune axis and vascular pathways. Chronic stress and negative affect have been identified as risk factors for BPPV, possibly due to the dysregulation of the hypothalamic–pituitary–adrenal (HPA) axis and subsequent hormonal fluctuations (Hsu et al. 2019; Z. J. Chen et al. 2016). Moreover, vascular integrity appears to play a crucial role; systemic conditions such as hypertension, diabetes, and hyperlipidemia—known to impact microcirculation—are significantly prevalent in patients with BPPV and are recognized risk factors for its occurrence (J. Chen et al. 2020; Song et al. 2023). Since ischemic events and vascular insufficiency are increasingly linked to vestibular pathology, the “vascular protection” conferred by high psychological well‐being may preserve macular integrity by mitigating these underlying vascular risks (Song et al. 2023).

### 4.3 Comparison With Previous MR Studies

While recent MR analyses have indicated a complex relationship where antidepressant use, rather than depression itself, shows a potential causal link to BPPV (Liao et al. 2025), our study utilized significantly larger GWAS datasets, such as those defining the well‐being spectrum (Okbay et al. 2016), and a broader range of phenotypes. This increased statistical power allowed us to detect nuanced inverse associations—such as the inverse effect of educational attainment on BPPV risk—that were less emphasized in prior investigations (Guo et al. 2025). Moreover, by confirming null results in the reverse direction (BPPV to mental health), which aligns with previous findings of no significant genetic association between BPPV and multiple psychiatric disorders (Liu et al. 2024), we clarify that the strong comorbidity observed in clinics is likely driven by shared environmental stressors or the immediate psychological impact of acute vertigo (Fox et al. 2019), rather than a direct genetic consequence of the vestibular disorder itself, consistent with the high prevalence of unrecognized psychiatric comorbidities in vertigo patients (Limburg et al. 2018).

### 4.4 Strengths and Limitations

The primary strength of this study is the triangulation design. However, significant limitations remain. The NHANES criteria may be susceptible to diagnostic overlap with other conditions causing movement‐triggered vertigo, such as vestibular migraine or orthostatic dizziness, increasing the risk of misclassification. Conversely, relying on ICD‐10 codes in the UK Biobank may capture only clinically diagnosed cases, potentially missing milder cases treated in primary care, thereby underestimating true incidence and introducing healthcare‐seeking bias. Furthermore, the outcome definitions varied significantly between NHANES (self‐reported questionnaire) and UK Biobank (ICD‐10 codes). Additionally, it is important to note that the effect estimates derived from different methods (ORs in NHANES/MR vs. HRs in UK Biobank) are not directly comparable due to fundamental differences in study designs and outcome measures; thus, the triangulation demonstrates directional consistency rather than magnitude equivalence. While MR provides evidence less prone to reverse causation, psychological traits are highly polygenic and inextricably linked with complex behavioral and socioeconomic factors. Consequently, genetic instruments may influence BPPV risk through pleiotropic pathways independent of the studied exposures (Slob and Burgess 2020). The relatively small number of SNPs for certain traits (e.g., four SNPs for emotional lability) also raises concerns regarding instrument strength. Finally, the GWAS participants were predominantly of European descent, warranting caution when generalizing findings to other diverse populations.

## 5 Conclusion

In conclusion, this multi‐database study provides consistent evidence suggestive of a potential relationship where psychological factors are not merely secondary bystanders in BPPV but may be active determinants of disease risk. We identified a potential inverse association of life satisfaction and neuroticism against the clinical onset of BPPV, supported by real‐world, longitudinal, and genetic data. These findings should not be interpreted as evidence for implementing psychological interventions to prevent BPPV or changing clinical practice at this stage. Rather, they identify an intriguing association that warrants further investigation and independent replication in prospective studies with clinically confirmed BPPV outcomes.

## Author Contributions

**Yingying Wang**: formal analysis. **Rong Yu**: writing – review and editing, conceptualization, investigation. **Kai Wang**: data curation. **Fengzhao Yang**: data curation. **Hongqun Jiang**: validation, project administration. **Wugen Luo**: conceptualization, formal analysis, writing – original draft, validation, writing – review and editing. **Siyi Li**: formal analysis.

## Funding

This work was supported by the National Natural Science Foundation of China Youth Program (grant no. 82101237); the General Project of Natural Science Foundation of Jiangxi Province (grant no. 20252BAC240583); the Key Project for Clinical Cultivation of The First Affiliated Hospital of Nanchang University (grant no. YFYLCYJPY202404); and the Key Clinical Specialty of Jiangxi Province (grant no. 202382).

## Ethics Statement

The observational components of this study utilized de‐identified, publicly available data from the NHANES and the UK Biobank. The NHANES protocol was approved by the National Center for Health Statistics Research Ethics Review Board, and all participants provided written informed consent. The UK Biobank study received ethical approval from the North West Multi‐Centre Research Ethics Committee (MREC), and all participants provided written informed consent at recruitment. The Mendelian randomization analysis used summary‐level data from published GWAS studies (FinnGen and others), which received ethical approval from their respective institutional review boards. As this study relied on secondary analysis of anonymized data, specific institutional ethics approval for this analysis was waived.

## Consent

The authors have nothing to report.

## Conflicts of Interest

The authors declare no conflicts of interest.

## Supporting information




**Table S1. Details of GWAS Datasets**. Summary of the data sources used for Mendelian Randomization, including consortium names (FinnGen, UK Biobank, etc.), sample sizes, number of SNPs, and population ancestry.
**Table S2. Full Mendelian Randomization Results (Forward and Reverse)**. Detailed statistical output for all MR methods (IVW, MR‐Egger, Weighted Median, Weighted Mode) for both the “Mental Health to BPPV” and “BPPV to Mental Health” analyses. Columns include beta coefficients, standard errors, p‐values, and heterogeneity statistics.
**Table S3. Sensitivity and Pleiotropy Analyses**. Results of the MR‐Egger intercept test for directional pleiotropy and the MR‐PRESSO global test for outliers. This table also lists the number of outliers removed and the corrected causal estimates after outlier removal.

**Figure S1. Reverse Mendelian Randomization Analysis**. Forest plot displaying the causal effect of genetic liability to BPPV on various mental health outcomes. The analysis used the IVW method. No significant associations were found for any mental health trait, supporting the directionality of the main findings from psychology to vestibular function.

## Data Availability

The datasets analyzed during the current study are publicly available. NHANES data can be downloaded from the CDC website (https://www.cdc.gov/nchs/nhanes/). UK Biobank data are available to researchers upon application (https://www.ukbiobank.ac.uk/). Summary statistics for the Mendelian randomization analysis were obtained from the FinnGen study (https://r5.risteys.finngen.fi/) and the GWAS Catalog (https://www.ebi.ac.uk/gwas/).
