# Supplementary material for: Psychological Well‐Being, Neuroticism, and the Risk of Benign Paroxysmal Positional Vertigo: A Triangulation Study
Source: Brain Behav. 2026 Jul 8;16(7):e71569. doi: 10.1002/brb3.71569 (PMC13344896; doi:10.1002/brb3.71569)
Supplement: Supplementary file 1 — Table S1. Details of GWAS Datasets. Summary of the data sources used for Mendelian Randomization, including consortium names (FinnGen, UK Biobank, etc.), sample sizes, number of SNPs, and population ancestry. Table S2. Full Mendelian Randomization Results (Forward and Reverse). Detailed statistical output for all MR methods (IVW, MR‐Egger, Weighted Median, Weighted Mode) for both the “Mental Health to BPPV” and “BPPV to Mental Health” analyses. Columns include beta coefficients, standard errors, p‐values, and heterogeneity statistics. Table S3. Sensitivity and Pleiotropy Analyses. Results of the MR‐Egger intercept test for directional pleiotropy and the MR‐PRESSO global test for outliers. This table also lists the number of outliers removed and the corrected causal estimates after outlier removal. [file BRB3-16-e71569-s002.docx]

**Table S1. Details of GWAS datasets used in the Mendelian randomization analysis.**

| **Phenotype** | **Source / Consortium** | **Year** | **Study ID / Accession Code** | **Ancestry** | **Sample Size (N)** | **Cases (n)** | **Controls (n)** | **SNPs (n)** |
| --- | --- | --- | --- | --- | --- | --- | --- | --- |
| **Outcome** |  |  |  |  |  |  |  |  |
| Benign Paroxysmal Positional Vertigo (BPPV) | FinnGen (R5) | 2020 | Finngen_R5_H8_BPV | European | 213,416 | 3,834 | 209,582 | 16,380,466 |
| **Exposures (Mental Health)** |  |  |  |  |  |  |  |  |
| Life Satisfaction | GWAS Catalog | 2019 | GCST007337 (PMID: 30643256) | European | 80,852 | NA | NA | 2,336,260 |
| Positive Affect | GWAS Catalog | 2019 | GCST007338 (PMID: 30643256) | European | 410,603 | NA | NA | 2,336,260 |
| Neuroticism | GWAS Catalog | 2019 | GCST007339 (PMID: 30643256) | European | 523,783 | NA | NA | 2,336,260 |
| Emotional Lability | GWAS Catalog | 2021 | GCST90013452 (PMID: 33590662) | European | 3,268 | NA | NA | 7,569,228 |
| Well-being Spectrum | GWAS Catalog | 2019 | GCST007341 (PMID: 30643256) | European | 2,083,151 | NA | NA | 2,336,260 |
| Irritable Mood | GWAS Catalog | 2018 | GCST006941 (PMID: 29500382) | European | 366,726 | NA | NA | 10,894,596 |
| Frailty | Figshare | 2023 | PMID: 36928559 | European | 386,565 | NA | NA | NA |
| Leisure/Social Activities | IEU OpenGWAS | 2018 | ukb-b-4667 | European | 461,369 | 67,877 | 393,492 | 9,851,867 |
| Suffer from 'Nerves' | IEU OpenGWAS | 2018 | ukb-b-19957 | European | 445,809 | 94,665 | 351,144 | 9,851,867 |

*Abbreviations: GWAS, Genome-wide association study; SNP, Single nucleotide polymorphism; NA, Not applicable (continuous trait).*

**Table S2. Sensitivity and pleiotropy analyses for the Mendelian randomization estimates of mental health traits on BPPV.**

| **Exposure** | **Outcome** | **Heterogeneity (Cochran's Q)** | | **Pleiotropy (MR-Egger Intercept)** | | **MR-PRESSO Global Test** | |
| --- | --- | --- | --- | --- | --- | --- | --- |
|  |  | **Q Statistic** | **P-value** | **Intercept** | **P-value** | **RSSobs** | **P-value** |
| Emotional Lability | BPPV | 3.00 | 0.114 | -0.012 | 0.485 | 4.21 | 0.0485* |
| Frailty | BPPV | 135.00 | 0.114 | 0.003 | 0.672 | 140.5 | 0.231 |
| Irritable Mood | BPPV | 121.00 | 0.580 | 0.001 | 0.891 | 125.3 | 0.610 |
| Leisure/Social Activities | BPPV | 152.00 | 0.692 | -0.002 | 0.745 | 155.8 | 0.712 |
| Life Satisfaction | BPPV | 268.00 | 0.502 | 0.008 | 0.023* | 275.4 | 0.422 |
| Suffer from 'Nerves' | BPPV | 103.00 | 0.346 | 0.005 | 0.512 | 108.2 | 0.389 |
| Neuroticism | BPPV | 382.00 | 0.080 | 0.001 | 0.556 | 395.1 | 0.032* |
| Positive Affect | BPPV | 332.00 | 0.339 | 0.002 | 0.612 | 341.5 | 0.365 |
| Well-being Spectrum | BPPV | 416.00 | 0.247 | 0.003 | 0.489 | 428.9 | 0.281 |

*Abbreviations: MR-PRESSO, Mendelian Randomization Pleiotropy RESidual Sum and Outlier; RSSobs, Residual Sum of Squares Observed. * Indicates P < 0.05, suggesting potential pleiotropy or outliers. Outliers were removed where MR-PRESSO was significant.*

**Table S3. Full Mendelian randomization results for the bidirectional association between mental health traits and BPPV.**

| **Exposure** | **Outcome** | **Method** | **NSNP** | **Odds Ratio (OR)** | **95% CI Lower** | **95% CI Upper** | **P-value** |
| --- | --- | --- | --- | --- | --- | --- | --- |
| **A. Association of Mental Health Traits with BPPV Risk (Forward Analysis)** | | | | | | | |
| Emotional Lability | BPPV | IVW (FE) | 4 | 0.788 | 0.643 | 0.967 | 0.0228* |
| Frailty | BPPV | IVW (FE) | 136 | 1.334 | 0.879 | 2.026 | 0.1760 |
| Irritable Mood | BPPV | IVW (FE) | 122 | 0.956 | 0.677 | 1.352 | 0.8000 |
| Leisure/Social Activities | BPPV | IVW (FE) | 153 | 1.065 | 0.389 | 2.912 | 0.9030 |
| Life Satisfaction | BPPV | IVW (FE) | 269 | 0.544 | 0.370 | 0.800 | 0.0020* |
| Suffer from 'Nerves' | BPPV | IVW (FE) | 104 | 2.626 | 0.941 | 7.330 | 0.0652 |
| Neuroticism | BPPV | IVW (FE) | 383 | 0.692 | 0.521 | 0.921 | 0.0115* |
| Positive Affect | BPPV | IVW (FE) | 333 | 0.625 | 0.426 | 0.916 | 0.0161* |
| Well-being Spectrum | BPPV | IVW (FE) | 417 | 0.594 | 0.378 | 0.934 | 0.0241* |
| **B. Association of BPPV with Mental Health Traits (Reverse Analysis)** | | | | | | | |
| BPPV | Emotional Lability | IVW (FE) | 6 | 0.995 | 0.839 | 1.180 | 0.9510 |
| BPPV | Frailty | IVW (FE) | 9 | 0.999 | 0.990 | 1.008 | 0.8600 |
| BPPV | Irritable Mood | IVW (FE) | 9 | 1.007 | 0.995 | 1.019 | 0.2410 |
| BPPV | Leisure/Social Activities | IVW (FE) | 10 | 1.001 | 0.997 | 1.004 | 0.7240 |
| BPPV | Life Satisfaction | IVW (FE) | 5 | 0.997 | 0.986 | 1.007 | 0.5250 |
| BPPV | Suffer from 'Nerves' | IVW (FE) | 10 | 0.996 | 0.992 | 1.000 | 0.0733 |
| BPPV | Neuroticism | IVW (MRE) | 5 | 0.996 | 0.979 | 1.014 | 0.6540 |
| BPPV | Positive Affect | IVW (FE) | 5 | 0.996 | 0.987 | 1.005 | 0.4130 |
| BPPV | Well-being Spectrum | IVW (MRE) | 5 | 0.998 | 0.987 | 1.009 | 0.6740 |

*Abbreviations: IVW, Inverse Variance Weighted; FE, Fixed Effects; MRE, Multiplicative Random Effects; NSNP, Number of Single Nucleotide Polymorphisms; CI, Confidence Interval. * Indicates statistically significant results (P < 0.05).*
