# Supplementary figures and images for: Psychological Well‐Being, Neuroticism, and the Risk of Benign Paroxysmal Positional Vertigo: A Triangulation Study
Source: Brain Behav. 2026 Jul 8;16(7):e71569. doi: 10.1002/brb3.71569 (PMC13344896; doi:10.1002/brb3.71569)

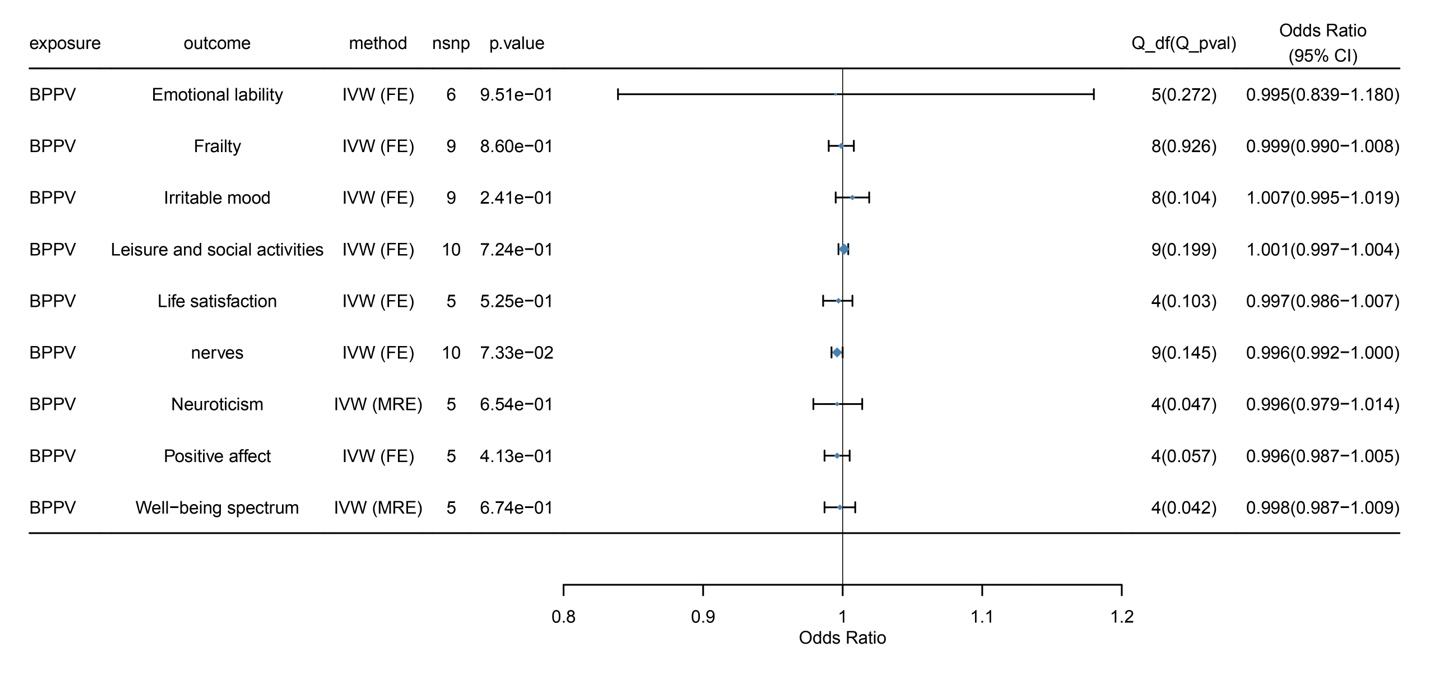

Supplement: Supplementary file 2 — Figure S1. Reverse Mendelian Randomization Analysis. Forest plot displaying the causal effect of genetic liability to BPPV on various mental health outcomes. The analysis used the IVW method. No significant associations were found for any mental health trait, supporting the directionality of the main findings from psychology to vestibular function. [file BRB3-16-e71569-s001.jpg]
